# MIS-C and co-infection with *P. vivax* and *P.* *falciparum* in a child: a clinical conundrum

**DOI:** 10.1186/s13052-022-01311-9

**Published:** 2022-07-27

**Authors:** Michela Scalisi, Salvatore Giordano, Laura Antonella Canduscio, Maria Concetta Failla, Luca Messina, Elisa Sferrazza, Raffaella Rubino, Lucia Siracusa, Veronica Vanella, Antonio Cascio, Claudia Colomba

**Affiliations:** 1 Division of Paediatric Infectious Diseases, “G. Di Cristina” Hospital, ARNAS Civico, Palermo, Italy; 2 Department of Health Promotion, Maternal and Infant Care, Internal Medicine and Medical Specialties, University of Palermo, Palermo, Italy

**Keywords:** Case report, Multisystem inflammatory syndrome, SARS CoV2, Malaria, COVID-19

## Abstract

**Background:**

The ongoing Coronavirus Disease 2019 (COVID-19) epidemic represents an unprecedented global health challenge. Many COVID-19 symptoms are similar to symptoms that can occur in other infections. Malaria should always be considered in patients with SARS-CoV-2 infection returning from endemic areas.

**Case presentation:**

We present the first case of multisystem inflammatory syndrome (MIS-C) and Plasmodium vivax-falciparum and SARS-CoV2 coinfection in children. Despite clearance of parassitaemia and a negative COVID-19 nasopharyngeal PCR, the patient’s clinical conditions worsened. The World Health Organization (WHO) criteria were used to make the diagnosis of MIS-C. Treatment with intravenous immunoglobulins and methylprednisolone was effective.

**Conclusions:**

This case emphasizes the importance of considering malaria diagnosis in patients returning from endemic areas, even in the COVID 19 era. Malaria and SARS-CoV2 co-infection may increase the risk of MIS-C, for which early detection is critical for proper management.

## Background

The ongoing Coronavirus Disease 2019 (COVID-19) epidemic represents an unprecedented global health challenge. Currently, health systems around the world are enduring unparalleled efforts, and physicians continue to play a critical role in early detection and clinical management of COVID-19 pandemic [1]. Many COVID-19 symptoms, such as fever, myalgia, and headache, are similar to symptoms that can occur in other infections and an appropriate epidemiological approach and differential diagnosis are critical for selecting the appropriate clinical intervention [2, 3]. Despite the fact that the spread of COVID-19 in Africa has been slower than in other areas, 3 million cases and over 78,000 deaths due to COVID-19 have been recorded. Furthermore, African countries account for roughly 94% of all malaria cases and deaths worldwide. Malaria should always be considered in patients with SARS-CoV-2 infection returning from endemic regions due to overlapping geographical pathogens causing the COVID-19 and malaria co-infections and because the two clinical conditions resemble each other [4, 5]. However, there is a significant knowledge gap regarding the coexistence of these two diseases, particularly in children [6, 7].

We present the first case of *falciparum* and *vivax* malaria, as well as concurrent SARS-CoV-2 infection in an 8-year-old girl from Ivory Coast with the multisystem inflammatory syndrome in children (MIS-C). The case reminds us how important it is considering the patient’s history, travel history, and epidemiologic context even during this coronavirus pandemic.

## Case presentation

An Italian 8-year-old girl from Ivory Coast presented to our hospital’s emergency department with a three-day fever, inappetence, and abdominal pain. Her previous medical history revealed risperidone-treated autism; she had no prior contact with a COVID 19-confirmed case. Her parents had received COVID 19 vaccination, but because her clinical picture was compatible with COVID-19, she was admitted to our department for a nasal swab for SARS-CoV2.

Due to the patient’s autistic condition, our physical examination was made more difficult; as far as we could tell, the child was conscious but in poor clinical conditions. Mild signs of dehydration and bilateral eyelid oedema, pharyngeal hyperaemia, and posterior cervical lymph node hypertrophy were noted. A respiratory examination revealed rhonchi. An abdominal examination showed mild hepatomegaly. The cardiovascular and neurological examination were both normal.

The laboratory workup revealed elevated inflammatory parameters (C-reactive protein, CRP 8.4 mg/dL, procalcitonin 2.2 μg/L, ferritin 1070 μg/L), bilirubin (total bilirubin 2.19 mg/dL, indirect bilirubin 1.2 mg/dL), lactate dehydrogenase (LDH) 716 U/L and transaminases (alanine transaminase, ALT 89 U/L and aspartate transaminase, AST 100 U/L). Her total blood count showed a low platelet count 4390/uL. A nasopharyngeal swab was positive for SARS-CoV2 polymerase chain reaction (PCR) as antibody IgG against SARS-CoV2.

Broad-spectrum antibiotic therapy was initiated with endovenous ceftriaxone.

During a detailed history in our infectious disease department, the mother reported that she was taking mefloquine hydrochloride for malaria prophylaxis for a recent trip to Africa with her daughter.

**Fig. 1:**
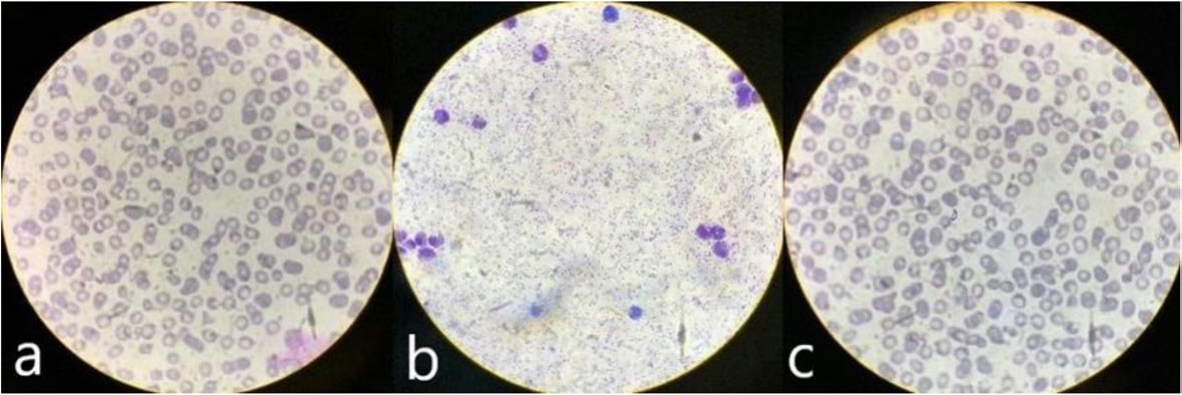
**a**. Trofozoite falciparum revealed at thin smear; **b**. Trofozoite falciparum revealed at thick blood smear; **c**. Schizonte vivax revealed at thin smear

Therefore, thick and thin blood smear, rapid antigen test, and PCR for malaria were performed urgently, and the girl was diagnosed with malaria by *Plasmodium falciparum and Plasmodium vivax* with a parasitaemia of 5% (Fig. 1). After ruling out glucose 6 phosphate dehydrogenase deficiency, specific drug treatment with atovaquone-proguanil for a total of 3 days, followed by 14 days of primaquine, was started.

Despite repeated malaria smear after 5 days from initial presentation revealed clearance of parasitaemia and a COVID-19 nasopharyngeal PCR repeated 7 days after admission was confirmed as negative, the patient’s clinical conditions worsened. Fever was persistent as did the abdominal pain and a diffuse erythematous rash appeared. Coinfections with *Neisseria meningitidis, Haemophilus influenzae, Streptoccoccus pneumoniae, Streptoccoccus agalactiae, Klebsiella pneumoniae, Listeria monocytogenes, Escherichia coli* were ruled out.

**Fig. 2:**
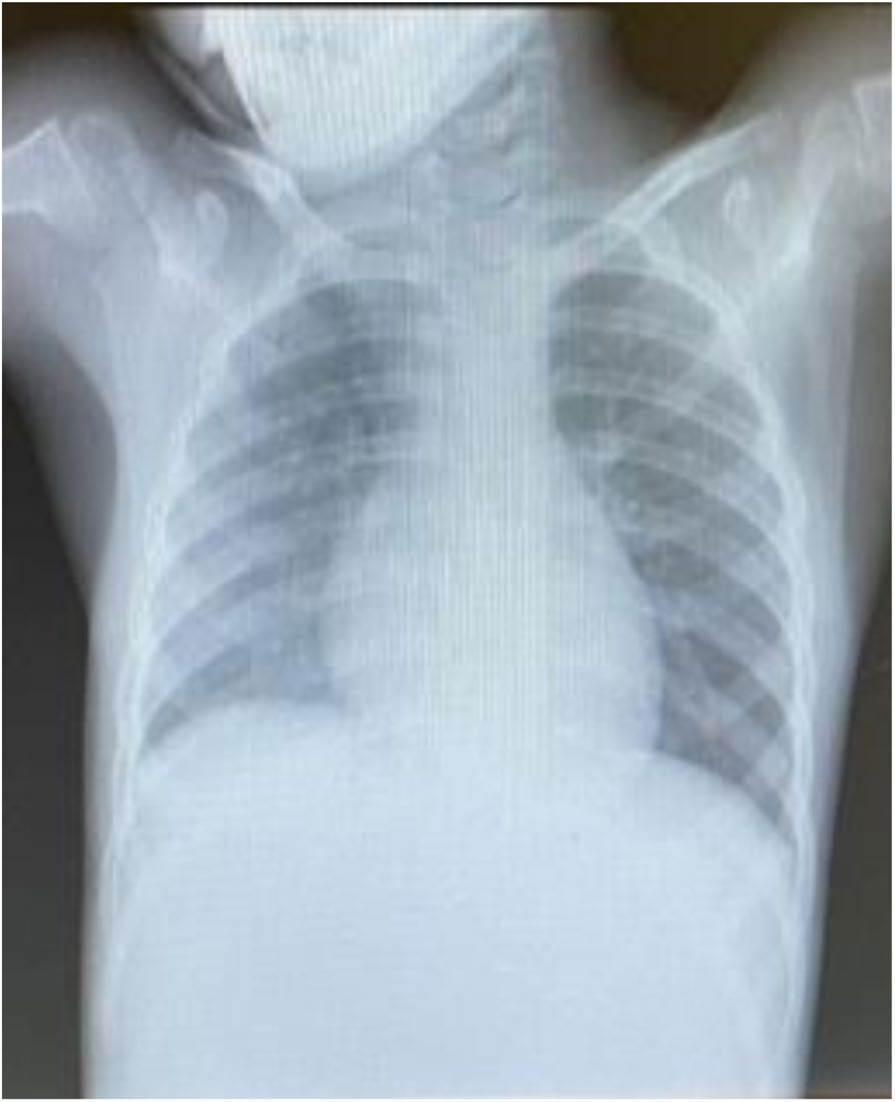
Chest X-ray. Hypo transparency of the right lung fields with accentuation of micronodular reticular pulmonary character pattern, especially on the right side

**Fig. 3:**
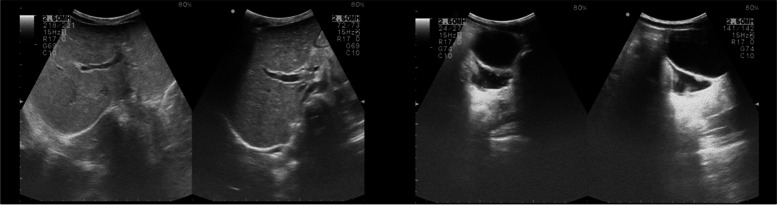
Abdomen ultrasound. Hepatomegaly with starry sky appearance, pelvic intraperitoneal fluid

Further laboratory investigations were performed to document organ damage in accordance with guideline diagnostic criteria in cases where MIS-C was suspected. Cardiovascular involvement was investigated by analysis of troponin and pro B-type natriuretic peptide (ProBNP) values, respectively 8.3 ng/L and 520 pg/mL. Therefore, electrocardiogram, which was normal, and cardiac ultrasound, which showed mild mitral insufficiency, were performed. A chest X-ray (Fig. 2) showed accentuation of micronodular reticular pulmonary character pattern; ultrasound of the abdomen revealed hepatomegaly with starry sky appearance, as well as peritoneal fluid (Fig. 3).

The World Health Organization (WHO) criteria were used to make the diagnosis of MIS-C [7]. Therapy with a single dose of intravenous immunoglobulins (IVIG) at a dosage of 2 g/Kg and intravenous methylprednisolone at a dosage of 30 mg/Kg/day was started in accordance with guidelines, and performed for three days, followed by methylprednisolone at a dosage of 2 mg/Kg/day.

Acute anaemia was treated with a blood transfusion.

The treatment was well tolerated and quickly effective, resulting in an overall improvement in the patient’s clinical conditions. The child became apyretic after administration of immunoglobulin and the first bolus of methylprednisolone. Blood tests gradually returned to normal (Table 1).

**Table 1:** Laboratory results

Laboratory Tests	1th day	3rd day	6th day	Reference Range
White blood cell count	7.430	12.240	12.610	5.4–9.7 10^3^/uL
Absolute neutrophil count	5.430	6.170	6.680	2.6–6 10^3^/uL
Lymphocytes	1.55	3.980	5.170	1.2–2.8 10^3^/uL
Haemoglobin	11.2	8.3	9	11.3–13.4 g/dL
Platelets	43.900	27.400	130.600	219–339 10^3^/uL
CRP	8.4	14.34	3	0–0.5 mg/dL
Procalcitonin	2.2	7.26	1.4	0.02–0.05 μg/L
Total Bilirubin	2.19	4.01	1.29	0–1 mg/dL
Bilirubin Direct	0.98	3.31	1.15	0–0.29
Indirect Bilirubin	1.20	0.7	0.14	0–0.90
Sodiemia	137	132	137	138–145 mmol/L
Albumin	3.7	2.8	3	3.8–5.4 g/dL
AST	100	76	47	15–55 U/L
ALT	89	62	46	5–33 U/L
Gamma-glutamil-transpeptidasi (gGT)			144	5–32 U/L
LDH	716	718	619	135–214 U/L
Cholesterol	83	67		110–200 mg/dL
Triglycerides	296	476	445	35–114 mg/dL
Ferritin	1070	1846		10–140 μg/L
Interleukin 6 (IL6)	588	332		0–7 pg/mL
ProBNP	141	520		0–145 pg/mL
Troponin	5	8.3		0–14 ng/L

## Discussion

This is the first report of MIS-C and *Plasmodium vivax* and *falciparum* malaria co-infection. By presenting this case, we aimed to draw attention to a rare travel related infection masked by a pandemic infection. Indeed, since the end of 2019, the global outbreak of COVID-19 has spread rapidly posing a major challenge to many healthcare systems around the world [8]. In this situation, co-infections present a unique challenge to clinicians and it is essential to remember that neither co-infection with other pathogens can be ruled out when COVID-19 is confirmed, nor does a positive test for other pathogens completely negate the presence of co-infection with COVID-19.

This is especially true in malaria-endemic areas or among people who have recently returned from malaria-endemic countries. Malaria and COVID-19 can actually presents with fever, myalgia, fatigue, headache, gastrointestinal symptoms, and both can have a negative impact on the course of the other [9, 10]. Furthermore, the diagnosis of malaria is already elusive in a non-endemic country; this difficulty appears to be exacerbated by overlap of symptoms and signs with COVID-19. The similarity in the non-specific symptoms and febrile illness associated with COVID-19 and malaria makes missing a malaria diagnosis in the COVID-19 pandemic highly likely especially in country not endemic for malaria.

In our case, the patient was admitted due to COVID-19 and was found to have a concomitant *falciparum* and *vivax* malaria infection whose pattern of over-expressed cytokines overlaps with that of COVID-19. Malaria with severe manifestations may be caused by an elevated proinflammatory response (IL 6, IL 1, and tumor necrosis factor alpha increase during haemolysis from malarial infection). Therefore, coinfection with malaria and SARS-CoV2 may lead to an increased risk of developing MIS-C in paediatric age. Co-infection with a parasite and a virus, in particular, can place a double burden on the body’s immune system. Immune cells may undergo a dilemma of whether to produce a response to eliminate one pathogen or the other and ultimately end up with an exaggerated immune response. MIS-C is a novel dangerous and potentially life-threatening entity associated with a wide range of clinical features including persistent fever, digestive symptoms, rash, bilateral non purulent conjunctivitis, mucocutaneous inflammation signs, and frequent cardiovascular involvement. MIS-C is frequently associated with hemodynamic failure, with acute cardiac dysfunction requiring hemodynamic support in 60 to 75% of cases, sometimes leading to death [11]. We diagnosed our patient with MIS-C due to clinical deterioration characterized by continuous fever, a multisystem organ involvement with elevation of inflammatory markers, despite the clearance of parasitaemia after malaria treatment. Many altered biochemical parameters such as anaemia, thrombocytopenia, elevated bilirubin, CRP, procalcitonin, AST, ALT, ferritin, GGT, IL 6, and reduced sodium and albumin levels can be related to both malaria and MIS-C therefore require proper interpretation (Table 2). In severe malaria, there is generally a decline in red blood cell, haemoglobin, and platelet counts due to sequestration of infected red blood cells caused by the activation of endothelial activation markers and surface adhesion molecules. However, ours was not a case of severe malaria, but rather a case of malaria complicated by MIS-C in fact even SARS-CoV-2 infection is associated with a cytokine cascade and endothelial activation [12–14].

**Table 2:** Laboratory changes in patient with malaria and MIS-C

	MALARIA	MIS-C
Anaemia	X	X
Thrombocytopenia	X	X
Leukopenia or leukocytosis	X	X Leukocytosis with neutrophilia and lymphopenia
Increased CRP	X	X
Procalcitonin	X	X
Increased fibrinogen	X	X
Increased D-dimer		X
Increased LDH	X	X
Hypertransaminasemia	X	X
Hyperbilirubinemia	indirect	direct
Hyponatremia	X	X
Increased IL-6	X	X
Hypoglycemia	X	
Hyperferritinemia		X
Metabolic acidosis	X	X
Hypoalbuminemia		X
Increased cardiac enzymes		X

MIS-C management has evolved over the course of the pandemic. Current guidelines recommend IVIG and/or corticosteroids as first-line therapy. Other interventions are determined by the disease severity, outcome, and response to initial therapy. Antithrombotic therapy and second-line treatment with several immunomodulatory drugs (interleukin-1 inhibitor, interleukin-6 inhibitor), and other supportive therapeutic agents are used concurrently in these cases [15].

Single case reports of patients with malaria and COVID-19 co-infection have been described by several authors [16–18] and a recent systematic review reports concomitant infections of SARS-CoV-2 and malaria, mainly in adults (age range 4–67 years) [6]. There are very few cases of co-infection malaria-COVID described in the child. Adetola et al. described four children with COVID and malaria treated with a full course of anti-malaria medications [19]. Rashmi Kishore et al. reported one case of *P. vivax* malaria reactivation in an Indian 10-year-old boy suggesting a possible role of COVID-19 in inducing malarial relapse [20]. Although the exact mechanism causing this activation is unknown, the cytokine cascade associated with systemic disease could also induce reactivation of *Plasmodium vivax* from a previous infection with dormant liver stage parasites or hypnozoites. In our case we can’t say if it was the first malaria vivax infection or a case of malarial relapse because we don’t know if it was her first trip to Africa since she was born in Italy.

In none of the few cases of co-infection described to date COVID-19 has manifested itself in a severe form and required intensive support [21].

Ours is the first severe form of COVID-19 and malaria co-infection in the child due to the establishment of MIS-C. Regarding malaria treatment, the role of anti-malarials, e.g. artemisinin derivates and chloroquine, in the COVID-19 pandemic is complex. Artemisinin derivates, artemisinin-based combination therapy, and chloroquine have all been shown in vitro to be effective against SARS-CoV-2 [22, 23]. Several clinical trials, however, failed to confirm such a beneficial effect [24, 25].

## Conclusions

This case highlights how important is to consider the patient’s travel history, the epidemiologic setting and alternative diagnoses even under pandemic conditions [26]. Malaria should always be considered in patients who have recently returned from endemic areas. COVID-19 and malaria co-infection can be dangerous because the combination causes a cytokine storm, which is responsible for the more severe COVID-19 manifestations in children [14, 27, 28], such as MIS-C. As paediatric infectious disease physicians, it is our responsibility to stress the importance of the diagnosis of MIS-C in all patients with SARS-CoV2 infection despite the evidence of other etiological causes that could explain and confound the clinical case. Our case again demonstrates the importance of malaria prophylaxis. If the patient had received malaria prophylaxis during her trip, she probably would have been protected from malaria and would have had a less severe course of MIS-C.

## Data Availability

All data used and/or analysed during this study are included in this published article.

## Abbreviations

MIS-C: Multisystem Inflammatory Syndrome in Children
PCR: Polymerase Chain Reaction
SARS-CoV-2: Severe Acute Respiratory Syndrome Coronavirus 2
COVID-19: Coronavirus Disease 2019
LDH: lactate dehydrogenase
CRP: C-reactive protein
IL 6: nterleukin 6
AST: aspartate transaminase
ALT: alanine transaminase
